# Advances in Neurotransmission: The Remarkable Versatility of the Nervous System

**DOI:** 10.3390/ijms27156758

**Published:** 2026-07-28

**Authors:** Mario Manto, Jing-Ning Zhu, Hiroshi Mitoma

**Affiliations:** 1 Department of Neurology, Unité des Ataxies Cérébelleuses, Médiathèque Jean Jacquy, CHU-Charleroi, 6000 Charleroi, Belgium; 2 Department of Neurosciences, Université de Mons, 7000 Mons, Belgium; 3 State Key Laboratory of Pharmaceutical Biotechnology, National Resource Center for Mutant Mice, Institute for Brain Sciences, School of Life Sciences, Nanjing University, Nanjing 210023, China; 4 Department of Medical Education, Tokyo Medical University, Tokyo 160-0023, Japan

Neurotransmission is a fundamental process of communication within the body. Without neurotransmission, the central and peripheral nervous system cannot process the information appropriately. Neurotransmitters are essential for cognition, emotions, movement, sensory perception, activities of the autonomic nervous system, hormonal regulation, and all the homeostatic mechanisms within the body. This Special Issue highlights the numerous fields of research around the theme of neurotransmission, from behavior, neuropeptides, innervation of the uterus, neurotoxic agents, novel routes of administration of drugs, to novel therapies. The remarkable versatility of the nervous system is highlighted.

Impulsivity is a behavioral trait characterized by an urge to act without thinking, as a result of a deficient inhibitory control [1]. Impulsivity has important consequences in the daily life of individuals and for society [2]). Impulsivity is common in various prevalent psychiatric disorders, such as obsessive–compulsive disorder, bipolar disorders, borderline disorders, ADHD and addictive disorders, and is seen as a transdiagnostic factor in psychiatry [3].

Both genetic and neurobiological mechanisms underlie impulsivity [2]. Neurotransmitters might play a key role in the pathogenesis, with an implication of the limbic cortico-striatal pathways [4,5]. In particular, it has been suggested that impulsivity is modulated by the activity of the serotoninergic/dopaminergic system, and a serotoninergic/dopaminergic dysfunction has been raised [6]. In victims of suicide, it has been demonstrated in post-mortem studies that pre-synaptic 5-HT transporter (5-HTT) binding and post-synaptic 5-HT2 receptor binding are reduced. A dysfunction of the serotonergic system would, for instance, explain the impulsive aggression in dogs [7]. Kim et al. have investigated serotonergic activity in impulsivity traits by applying in vivo techniques in healthy subjects assessed by the Barratt Impulsiveness Scale [8]. Using MRI and PET, they show that motor impulsiveness is correlated with 5-HT availability. Non-planning impulsivity was correlated with 5-HT2A receptor availability in the fronto-striatal circuits, amygdala, and insula. These results emphasize the importance of these regions of the brain in impulsivity [4].

Our personality is shaped by complex interactions between our genetic background and the environment [9]. The field of behavioral epigenetics has modified our understanding of the factors influencing personality [10]. Amongst the genes influencing our personality are those of the serotoninergic system, and it is established that genetic variants modulate this neurotransmission [11].

Lachowicz et al. have investigated the relationships between 5-HTTLPR (serotonin transporter-linked promoter region; serotonin transporter gene: SLC6A4) polymorphisms and personality traits [12]. They found significant associations between polymorphisms and neuroticism, openness, and conscientiousness. The study shows that 5-HTTLPR polymorphisms are involved in human personality. Genetic variations in the SLC6A4 gene tune personality.

The cerebellum contains more than 20 neuropeptides, and it is assumed that they exert a modulatory function in the cerebellar circuitry and tune the development of the cerebellum [13,14]. For instance, CRF is found in climbing fibers, and its release is needed for motor learning at the parallel fiber-Purkinje cell synapse and motor coordination of cerebellar nuclei [14,15]. Rats injected with CRF directly into the cerebellum show superior motor performance [16].

Li et al. have reviewed 8 key neuropeptides (including BDNF, CRF, neuropeptide Y, orexin, oxytocin, secretin), discussing their potential therapeutic roles for disorders involving the cerebellar circuitry or for the modulation of neurological/psychiatric disorders [17]. Overall, neuropeptides might be involved in several neurological or neuropsychiatric disorders.

The innervation of the human uterine cervix has received little attention. The cervix has a sensory, parasympathetic, and sympathetic innervation [18]. Nerve fibers show a centripetal path from the serosa to the endometrium, and innervation of the myometrium is rich [19]. The innervation is denser in the supra-vaginal cervix than in the body of the uterus.

Malvasi et al. have studied the presence of catecholamine neurofibers in the pregnant uterus [20]. They found higher concentrations of A nerve fibers in the internal uterine orifice as compared to the lower uterine segment. These fibers might act as a myometrial pacemaker. The sympathetic system might be activated by stressful conditions and contribute to dystocic labor.

As compared to oral administration or the intravenous route, the nose-to-brain delivery of drugs has major potential for the management of brain disorders. Small molecules may be delivered by the intranasal route to reach the central nervous system [21]. Drug-carriers must be biocompatible and show low toxicity [22]. Drug-carrying nanoparticles may be delivered via the mouth or the nose, with increased therapeutic effects as compared to systemic delivery, with a bypass of the blood–brain barrier [23,24]. Nanoemulsion technologies are emerging [24].

A study of intranasal administration of indocyanine green (ICG) shows transport via the olfactory and trigeminal nerves [22]. ICG-encapsulated PLGA nanoparticles are potential carriers for many substances that could be useful for neurological diseases. Intranasal administration of a dry powder could be used to cross the nasal barrier.

Glyphosate, an organic phosphonate, is a widely used herbicide with broad-spectrum activity. The molecule accumulates in the kidney, liver and gastrointestinal tract wall [25,26,27,28]. The mechanisms of neurotoxicity include altered neurotransmission, neuroinflammation, oxidative stress, and mitochondrial dysfunction [29]. Neuronal death results from autophagy, necrosis, or apoptosis.

Palus et al. show that oral exposure to glyphosate impairs the neurochemical profile of the enteric nervous system in the porcine jejunum in a dose-dependent manner [30], 2025). After 28 days of exposure, glyphosate increases the number of neurons expressing PACAP, CGRP, CART and nNOS, but decreases the VAChT-positive neurons. Effects are broadly uniform. The authors highlight the need to reassess regulatory guidelines. Doses leading to neurotoxic effects are lower than the limits established by regulatory agencies [29].

The Zika virus infection is a mosquito-borne virus causing severe neurodevelopmental disorders (Zika congenital syndrome: decreased thickness of cortical layers, brain calcifications, gliosis, ocular lesions) and Guillain-Barré syndrome, being a threat to public health [31,32,33,34]. The impaired autoimmunity is likely due to molecular mimicry [35]. The expression of genes involved in neurotransmission is impaired. Maternal Zika infection, even inapparent, is associated with increased synaptic density in the hippocampus [36]. Microglial activation is associated with synaptic loss [37].

Rengifo et al. have investigated how the Zika virus impairs the GABA/Glutamate system in mice [38]. In the final phase of the infection, data suggest that a loss of glutamate and GABA synthesis is increased, promoting viral replication by an immunosuppressive effect and contributing to the synaptopathy.

With the aging of the population, cognitive impairment is a matter of concern on a global scale [39,40]. The search for novel drugs enhancing memory remains an important topic of research [41]. Astragaloside (extracted from Astragalus mongholicus) inhibits acetylcholinesterase. The molecule reduces memory impairment induced by scopolamine, being potentially a memory-enhancing drug given the importance of the cholinergic system in memory [42]. The drug does not reduce the memory impairment triggered by LPS, suggesting that astragaloside does not reverse neuroinflammation. The molecule shows a protective effect on hippocampal neurons via an effect on the PPARgamma/BDNF pathway [43].

Over the past four decades, the study of neurotransmitters has undergone substantial conceptual evolution. Initial investigations were devoted to establishing peptides and related molecules as bona fide neurotransmitters. With the subsequent rise in molecular biology, the field shifted toward the identification and classification of receptor families, and later toward elucidating the relationships between receptor function and synaptic plasticity.

The contributions assembled in this Special Issue indicate that neurotransmitter research is now entering a further stage of development. As outlined above, neurotransmitters are increasingly being examined through their associations with behavior, pathological processes, and the modulation of neural function—perspectives that extend beyond traditional molecular or synaptic frameworks.

Taken together, the articles in this issue demonstrate the broadening scope of neurotransmitter research. They elucidate links between neurotransmitter systems and impulsivity or personality traits, reproductive physiology, alterations in neurotransmitter profiles induced by viral infection or toxic exposure, therapeutic modulation of cerebellar and cognitive functions, and drug delivery to the brain via the nasal route.

Collectively, these studies underscore that the field is advancing toward a more integrative phase in which chemical signaling is considered not only at the molecular level but also as a determinant of behavior, physiology, and disease. We trust that readers will recognize from this collection that neurotransmitter research has entered a new and consequential stage of scientific inquiry.

## Data Availability

No new data were created or analyzed in this study. Data sharing is not applicable to this article.
